# What influences patients’ experiences of cannulation for haemodialysis performed by nursing staff? A qualitative interview study

**DOI:** 10.1177/17449871261466773

**Published:** 2026-09-18

**Authors:** Catherine Fielding, Sarah Brand, Louise Bramley, Maarten Taal, Caitlin Sorrell, Nicholas M Selby, Heather Buchanan

**Affiliations:** Lead Author, Advanced Practice Lead for Research and Innovation, Nottingham University Hospitals NHS Trust, Nottingham, UK; Advanced Clinical Practitioner, Healthcare of Older People, Nottingham University Hospitals NHS Trust, Nottingham, UK; Honorary Associate Professor, University of Nottingham, Nottingham, UK; Assistant Director of Nursing and Professions, Nottingham University Hospitals NHS Trust, Nottingham, UK; Assistant Director of Nursing and Deputy Clinical Director of Research and Innovation, Nottingham University Hospitals NHS Trust, Nottingham, UK; Professor of Medicine, Centre for Kidney Research and Innovation, School of Medicine, University of Nottingham, Nottingham, UK; Consultant Nephrologist, University Hospitals of Derby and Burton NHS Foundation Trust, Derby, UK; Clinical Associate in Psychology, Derbyshire Community Health Services NHS Foundation Trust, Ash Green Learning Disability Centre, Ashgate, Chesterfield, UK; Professor of Nephrology, Centre for Kidney Research and Innovation, School of Medicine, University of Nottingham, Nottingham, UK; Consultant Nephrologist, University Hospitals of Derby and Burton NHS Foundation Trust, Derby, UK; Professor, Lifespan & Population Health, School of Medicine, University of Nottingham, Nottingham, UK

**Keywords:** canulation, end-stage kidney disease, haemodialysis, nursing, patients’ experiences

## Abstract

**Background:**

Haemodialysis is a life-sustaining procedure for people with kidney failure, requiring cannulation with two needles before each treatment. Individuals undergo haemodialysis at least three times a week, leading to at least 312 cannulations each year. Some people find cannulation distressing and associated with noxious symptoms.

**Aim:**

This study aimed to explore what influences patients’ experiences of cannulation for haemodialysis as undertaken by nursing staff, generating understanding about improvements in procedure.

**Methods:**

Thirty participants (*n* = 30) from two renal centres in the United Kingdom completed semi-structured interviews. Intensive interviewing, constant comparison analysis and theoretical sampling from grounded theory were employed to meet the study aims.

**Results:**

Three categories describe the components that influence patients’ experiences of cannulation for haemodialysis: ‘Trying to make cannulation more comfortable’; ‘Preserving humanity and individuality’; and ‘The necessity of cannulation for haemodialysis forces coping’.

**Conclusion:**

Coping with cannulation for haemodialysis is dynamic with various facilitative components. Patients require strategies to make cannulation more comfortable, trust and empathy, and understanding and support to navigate and accept the challenges of cannulation.

## Plain Language Summary

Haemodialysis is a procedure for people whose kidneys no longer work, replacing their kidney function and keeping them alive. This happens three times a week for years. For most people, this requires the insertion of two needles into an enlarged or artificial vein. This needle insertion can be distressing. Our study aimed to explore what influences patients’ experiences of the needle insertion for haemodialysis, focussing on needle insertions performed by nursing staff. We completed 30 remote interviews with people on regular haemodialysis undergoing regular needle insertion. We analysed data alongside completion of interviews, using the findings to guide future interviews. This allowed us to explore concepts as they developed.

Our final analysis of the interviews identified three categories that summarise what influences patients’ experiences of the needle insertion for haemodialysis: (1) ‘Trying to make the needle insertion more comfortable’; (2) ‘Preserving humanity and individuality’; (3) ‘The necessity of the needle insertion for haemodialysis forces coping’. We found that coping with cannulation is dynamic and facilitated by components of the three categories. Various strategies made cannulation more comfortable, but further research is needed to evaluate how and if they work. Haemodialysis teams and training of these teams need to consider how to individualise needle insertion for haemodialysis and promote trust and empathy. Equipping patients and those who insert the needles to promote acceptance, avoiding stoicism, will improve individual experiences of this procedure.

These findings will allow us to consider how to improve patients’ experiences of needle insertion for haemodialysis, providing information for healthcare guidelines.

## Introduction

Haemodialysis is a treatment for end-stage kidney disease, replacing the functions of the kidneys (Fielding, 2024). As haemodialysis does not improve kidney function, patients can remain on haemodialysis for long periods of time, often years. Haemodialysis is required at least three times a week, to ensure fluid and electrolytes do not accumulate to dangerous levels, although the frequency of treatments can vary. Haemodialysis can be performed by the person themselves with support of carers, often in their own home. However, it mostly occurs in haemodialysis centres, performed by nursing and support staff.

Haemodialysis requires ‘artificial’ access to the circulation to perform the dialysis process on the blood whilst outside the body. A significant number of patients requiring haemodialysis use arteriovenous access for this purpose, either an arteriovenous fistulae comprising an ‘arterialised’ native vein, or an arteriovenous graft comprising an artificial ‘vein’ inserted between an artery and vein. All arteriovenous access requires two large needles to be inserted before each haemodialysis session, known as cannulation. Patients using an arteriovenous access for haemodialysis require at least 312 cannulations on 156 different occasions each year.

## Literature review

Although cannulation is a regular procedure for many patients, it is often distressing. The context of haemodialysis makes this a unique procedure, linked to a necessary treatment that happens repetitively over years (Fielding et al., 2023). Pain, anxiety, feelings of vulnerability and dependency, loss of control and worry about the success of cannulation are all negative emotions and beliefs linked with this procedure (Casey et al., 2014; Fielding et al., 2023; Mafara et al., 2016; Wilson and Harwood, 2017). Who performs the cannulation, where it happens and the patient’s perceived control over the procedure can affect patients’ experiences of cannulation (Casey et al., 2014; Fielding et al., 2023; Mafara et al., 2016; Wilson and Harwood, 2017).

Cannulation can be performed by the patient or carer, though the majority are performed by nursing staff. Self or carer cannulation has been explored in-depth in several individual studies (Fielding et al., 2023), with one study exploring what influences patients’ experiences in a mixed population of cannulation performed by self, carer and nursing staff (Moore et al., 2024). However, no study comprehensively explores what influences patients’ experiences of cannulation by nursing staff. Therefore, we performed an in-depth qualitative study to explore what influences patients perceived experiences of cannulation for haemodialysis by nursing staff, generating understanding for nursing staff, haemodialysis teams and policymakers about how to improve patients’ experiences of cannulation for haemodialysis.

## Methodology

This study adopted a qualitative approach drawing on the principles of grounded theory. However, not all elements of grounded theory were used, as the focus of the study was not to develop a theory. Constant comparison analysis (CCA) and intensive interviewing were used to add depth to the analysis (Charmaz, 2014; Chun Tie et al., 2019). What influenced experiences may not be fully formed within the participants’ conscious mind, requiring probing. The iterative process of CCA intensive interviewing aided the exploration of new insights as they occurred. The study protocol followed the ‘Standards for Reporting Qualitative Research’ (O’Brien et al., 2014) and was registered on the Research Registry (researchregistry7134). The protocol was designed with five patient representatives who assisted with the research aim and design and provided advice on the conduct of the study and analysis.

### Study setting and participants

Participants were recruited from two haemodialysis centres in the United Kingdom. We aimed to recruit up to 30 participants. This number was chosen to reflect the potential complexity of participants’ experiences, whilst managing the scope and timeframe of the study. Participants were 18+ years, undergoing cannulation of arteriovenous access by healthcare professionals for regular haemodialysis, including both English- and non-English-speaking people. Purposive sampling was used initially, followed by theoretical sampling (Chun Tie et al., 2019). Purposive sampling recruited 16 participants, to include participants who were both male and female with a mix of ethnicities, age, length of dialysis and cannulation and presence of diabetes (due to presence of vascular complications that may make cannulation more difficult). The final 14 participants were recruited using theoretical sampling, facilitating exploration of developing concepts (Supplmental Material 1).

### Data collection

Data were collected via semi-structured interviews conducted by the lead author alone using ‘Intensive interviewing’, where later interviews were adapted to explore developing concepts (Charmaz, 2014). An interview guide was developed and refined with patient representatives, using a previous systematic review as a guide (Fielding et al., 2023). This aimed to explore all potential concepts fully (Supplemental Material 2) and changed on two occasions (Supplemental Material 3) to respond to new insights congruent with intensive interviewing. All interviews were conducted remotely, due to restrictions in place for the COVID-19 pandemic, either via telephone or video call, with only the audio element of the interview recorded. One participant completed an interview using a medical interpreter. Brief notes were taken during the interview to ensure the interviewer was able to follow up on disclosures from the participant without interrupting the flow of the interview. Structured field notes were made immediately after each interview, guided by the notes taken during the interview, highlighting points of interest and events (Holloway and Galvin, 2017), as well as insights that could guide future interviews.

### Data analysis

Verbatim transcription of each interview was completed and checked for accuracy, re-familiarising the researcher with the interview. Transcripts of the interviews were uploaded into NVivo 12 for coding. Coding was conducted at three levels: Initial coding, Focussed coding and Theoretical coding (Charmaz, 2014; Chun Tie et al., 2019). These were not three separate phases but part of an iterative analysis that progressed backwards and forwards through these different levels of coding (Charmaz, 2014; Chun Tie et al., 2019). Initial coding was inductive, line-by-line and incident-by-incident. Focussed coding sorted through codes, refining and merging these and developing sub-categories and categories to summarise large portions of data. Links between categories were explored, considering what, when, where, why, how and consequences. Theoretical coding used diagrams to further refine the relationships between categories and codes to develop a complete story of the phenomenon.

Memos were used throughout the analysis to record the researcher’s reflections, developing further depth to the analysis (Charmaz, 2014; Chin Tie et al., 2019). The data analysis was completed by the lead author and discussed with co-authors and patient representatives throughout. Excerpts from interview transcripts, memos, codes, sub-categories and categories were used to facilitate this. Differences in opinions were discussed and led to further understanding of the data.

### Ethical considerations

The study received ethical approval from South Yorkshire Research Ethics Committee (REC No: 21/YH/0188) and the Health Regulatory Authority (IRAS No:300898). All participants provided face-to-face written informed consent to take part in the study, collected by the clinical team before the participant details were passed to the lead researcher to arrange the interview, with each participant given a copy of their consent form. Only the lead researcher could link the identity of the participant with their interview. All data were pseudo-anonymised before sharing with other research team members, with each participant given a participant identifier and allocated a random pseudonym for the analysis and reporting of the results. Verbal consent was checked prior to each interview.

In the event of participants becoming distressed, the interviewer paused the interview and ensured the participant was happy to continue. If a participant’s distress continued, with consent, they were to be referred to clinical teams for support, however this did not occur.

### Trustworthiness

Trustworthiness of the study was considered using Holloway and Galvin (2017). Thick description using participant voices was used throughout. Collaborators assisted with the analysis, including discussion with patient representatives. A reflexive diary (Holloway and Galvin, 2017) was kept by the lead researcher who was a haemodialysis nurse for 20 years prior to conducting this study to ensure that own opinions and assumptions did not unduly influence the research away from patients’ experiences (Fischer, 2009).

Following each interview, participants were provided with a summary description of their experiences of cannulation from haemodialysis, created from their interview. This was discussed during a follow-up telephone call, with the discussion recorded in a memo. This enabled participants to ‘member check’ the interpretation of their individual experience (Creswell and Poth, 2018). Where disagreement occurred (*n* = 1), this was discussed before inclusion in the analysis. Four participants were unable to review their interview summary.

## Results

Of the interviews, 27 were conducted via telephone and 3 via video call. The median interview time was 61 minutes (range: 30–79 minutes). Participants with a mix of demographic and clinical backgrounds were recruited (Table 1). A similar number of participants were recruited from each haemodialysis centre, with five participants from the second haemodialysis centre dialysing in a satellite unit. Participants ages ranged from 26 to 86 years old. Participants had a mix of lower (10, 33%) and upper arm arteriovenous access (20, 67%), but no-one with leg arteriovenous access. Participants had been undergoing cannulation for a range of periods from 5 months to 9 years. Nine participants used local anaesthesia prior to cannulation.

**Table 1.** Characteristics of participants undertaking semi-structured interviews.

Renal centre	
Renal centre 1	17 (57%)
Renal centre 2	13 (43%)
Main unit	25 (83%)
Satellite unit	5 (17%)
In a room on their own	4 (13%)
In a room with other patients	26 (87%)
Gender	
Male	16 (53%)
Female	14 (47%)
Age	
Median age (range)	55.5 (26.0–86.0) years
Ethnicity	
Caucasian	22 (73%)
Afro-Caribbean	4 (13%)
Indian or Pakistani	3 (10%)
Mixed ethnicity	1 (3%)
Median length of time on haemodialysis (this episode, without interruption)	27 (5–247 months)
Arteriovenous access type	
Lower arm fistula	10 (33%)
Upper arm fistula	18 (60%)
Upper arm grafts	2 (7%)
Used a central venous catheter previously for haemodialysis	
Yes	19 (63%)
No	11 (37%)
Type of access at start of haemodialysis	
Arteriovenous access	13 (43%)
Central venous catheter	16 (53%)
Unknown	1 (3%)
Cannulation technique	
Buttonhole	17 (57%)
Rope ladder	3 (10%)
Area puncture	10 (33%)
Length of time cannulating current arteriovenous access	
Median (range)	25 (2–118 months)
Less than 1 year	4 (13%)
1–2 years	10 (33%)
3–5 years	12 (41%)
More than 5 years	4 (13%)
Age of arteriovenous access at first cannulation	
Median (range)	3 (0–39) months
Less than 2 months	8 (27%)
2–6 months	15 (50%)
More than 6 months	7 (23%)
Local anaesthesia use	
None	21 (70%)
Sub-dermal lignocaine	5 (17%)
Topical anaesthetic cream–always	2 (7%)
Topical anaesthetic cream–occasionally	2 (7%)
Diabetes	
No	16 (53%)
Yes	14 (47%)
Vascular disease	
No	22 (73%)
Yes	8 (27%)

Overwhelmingly participants described throughout the interviews how difficult the cannulation process is. They went on to describe how this experience varied, and what contributed to making their experience either better or worse. Using CCA, these experiences formed 3 categories (which originated from 35 codes as part of the analysis process). These categories were:

• Trying to make cannulation more comfortable.
• Preserving humanity and individuality during cannulation.
• The necessity of cannulation for haemodialysis forces coping.

These categories were then further refined into sub-categories. The following section reports these findings using the three main categories enhanced with quotes highlighted in *italics*, using pseudo-anonymised names.

### Category 1: Trying to make cannulation more comfortable

Overwhelmingly, participant quotes used throughout this study describe how cannulation will never be a nice or pleasant procedure. Category 1 identifies how they try to make cannulation more comfortable. This is described below through four different sub-categories: familiarity, avoiding or minimising pain, increasing predictability and reducing anxiety. Throughout these sub-categories, participants also shared individual and practical hints and tips that helped them through cannulation. These are available in Supplemental Material 4 to help clinical practitioners and researchers identify and implement strategies that are important to individual patients and may be useful for further research and clinical recommendations.

Participants found cannulation alien, with cannulation being ‘*strange*’ (Emma). Developing ‘*familiarity*’ (Toby) with cannulation over time makes it easier. This is also about ‘*understanding a bit more about the process of the needles going in*’ (Charlotte) and ‘*so a lot of information would have been quite acceptable, better*’ (David). Participants describe how cannulation is painful, which they try to minimise or avoid: ‘*No-one wants to feel pain*’ (Nicholas). Participants describe using various strategies to minimise the pain from cannulation, including using local anaesthesia to help with pain, getting the needles in quickly and having a cannulator that causes less pain: ‘*If your top needlers are around they should be the ones to do your needling. It’s like if you have someone that’s going to hurt you and someone that isn’t*’ (Nicholas). Participants regularly describe how cannulation is variable and how and when it varies is unpredictable. This makes the cannulation harder to cope with: ‘*It was more of the mental side of it . . . it’s like a thin line, like, it could be right, could be wrong*’ (Nicholas).

If the cannulation goes well, participants want to minimise unpredictability by having: ‘*no changes, it should be as it is, now*’ (Graham). They often describe strategies to increase predictability (Supplemental Material 4). Different cannulators add to the unpredictability of cannulation, thus: ‘*if you could pick your nurse when you went in, who you want to needle you, you’d have the same nurse every time*’ (David).

Most participants experience anxiety about their cannulation and felt cannulation improved if they could reduce this anxiety: ‘*Eventually when I’d got relaxed and they could get the needles in easier, if you like, because I wasn’t so tense with it and that’s when the pain stopped*’ (Bridget).

Some describe how they have: ‘*learned how to . . . calm myself down a bit more now when I’m being needled . . . so it’s a lot easier*’ (Deborah). Participants often describe relaxing during cannulation as an active process, using different strategies to do this (Supplemental Material 4). Although it was not always easy, participants often describe distracting themselves where they have: ‘*that other option where you can probably, you know, think about or do something else rather than wait for the pain to come*’ (Toby).

### Category 2: Preserving humanity and individuality during cannulation

Participants indicate how their cannulation was: ‘*not just a physical process, it’s an emotional one as well I think*’ (Sarah). They highlight their humanity as: ‘*the mental aspect of a patient is most important . . . to ignore that is .. is bad . . . human beings as a corporate person, mind, physical bit and er.. they all go together*’ (George). Part of preserving their humanity is being treated as an individual: ‘*I think, looking at. . .um. . .personalised treatment and looking at my. . .er. . .my situation, my unique situation . . . I guess it’s a personal thing*’ (Simon).

The interaction and communication with the cannulator are key in preserving the participants’ individuality and humanity, creating: ‘*an interaction with that person, which just makes you feel a bit better*’ (Michael), as without this ‘*it makes you feel a bit worthless really . . . I feel out of control. I feel very upset and I feel angry*’ (Sarah). This is further described in two sub-categories exploring empathy and trust.

Participants describe how empathy from others makes needling easier and makes them feel not alone through cannulation: ‘*They were so kind . . . they understood what it’s going to feel like to stick the needle in me and they knew I was frightened*’ (Jessica).

Empathy from the cannulator was important to preserving their individuality and humanity:

*‘There’s got to be that personal element in it and ‘how are you? How are you doing? How are you feeling?’ . . . maybe that would be a bit of a reassurance on the person that they do care, you’re not on a conveyor belt . . . just five minutes or something like that, just to make that person think that you’re actually in their corner, that you’re actually thinking about them, which sounds a bit daft but I think it goes a long way’* (Michael).

Consequently, absence of empathy from the cannulator denied their individuality and humanity, making the cannulation: ‘*very impersonal . . . I felt like you were like cattle being put in and it wasn’t nice at all*’ (Toby).

Empathy could also come from family members or other patients:

*‘I had my partner with me every time I was needled, which kind of gave me a bit of reassurance . . . he didn’t, like, belittle me like I felt some of the staff did .. it was because he knew me’* (Deborah).

*‘Obviously it’s five chairs in a bay and we’re all talking about our own experiences what’s on dialysis when they’re needling . . . I think it makes me a little bit easier to accept what’s going off’* (David).

However, not all participants appreciate support from other patients, needing to be: ‘*mentally focused on myself*’ (Nicholas). Being able to trust the cannulator is important, as participants feel vulnerable through the cannulation: ‘*Well it means everything, like, to me, I see it as like the biggest bond .. like, my life is in their hands at that moment in time*’ (Nicholas). However, when things went wrong, they lost trust in the cannulator. If the dialysis unit was busy, this also undermined trust as it: ‘*makes you think what’s gone wrong today*’ (Graham) and ‘*it might be a bit of a rush job when they needle me*’ (Molly). See Table 2 for additional quotes.

**Table 2.** Quotes describing categories and sub-categories.

**Trusting the cannulator**
*‘So you’ve just got to face up to it and put your faith in the person that’s doing it haven’t you’.* (Joseph)
*‘I fully trust them what they do’* (Matthew).
*‘If it’s someone that I kind of trust and I know will do a good job, I think from the get-go I’m more relaxed about it’* (Clare).
*‘They know what’s best for me in the long run . . . I know they’re going to do their job and I know they do it as well to their capability . . . I always go by their judgement because in my eyes, as a patient .. well they know the complete ins and outs of how my veins could collapse . . . they won’t let anything bad happen’* (Rose).
**Loosing trust in the cannulator**
*‘Well I wondered whether they were sort of, like, competent or not! Sorry! . . . I know that sounds terrible but, you know, I thought oh, you know, obviously what are they doing if they can’t find the right fistula’* (Charlotte).
*‘I think if somebody had got an attitude and they’re sort of rushing their side of the job I’d probably think about it then’* (Bridget).
**The necessity of cannulation motivates participants to get through it**
*‘There is no choice but you get comfortable with it’* (Graham).
*‘I’ve got to get through–all I’ve got to get through the needling . . . if you haven’t got kidney failure, they’ll be ‘oh, I can’t do it, I won’t be able to do it’ but if you have to do it . . . you can do it’* (Jessica).
*‘It’s almost like breathing, isn’t it . . . It’s like this is what I’ve got to do now so it’s got to be done . . . all I did was tell myself it had to be done, yeah’* (Adrian).
*‘Well it’s rather like entering an aircraft which you know could drop down and once the door has shut, somehow your mind settles down to the inevitable and what has happened, you consent to it . . . It is at the present moment it’s not very pleasant but I have to go through it to have my dialysis’* (George).
**Cannulation occurs over long periods of time**
*‘I thought to myself ‘oh crikey I’ve got to go through this three times a week for the rest of my life’ and that was when the apprehension came in’* (Bridget).
*‘It’s OK having say done once, if you have it done three times a week, like, you know, if you like, forever and ever, you know, to an unknown specified date, you know, it’s a bit different isn’t it’* (Georgina).
*‘So regardless of whether that comes out and anything else comes off it, if I get a kidney or not, it’s always going to be that, you know. It’s always going to be needles and fistulas somewhere down the line . . . I’ve got to have this for all my life, for the rest of the days that I’m on this planet’* (Ben).
**Acceptance of unpleasant cannulation**
*‘It means accepting that I’m going to feel pain three times a week, but knowing that it won’t last long . . . I’m a bit more blasé now, I just think oh I’m going to be in pain and that’s the end of it!’* (Sarah).
*‘Because they change it every so often, it becomes an acceptance I think that they change. Your body accepts that, your arm accepts that and I think you get used to it’* (Daniel).
*‘You know, take it how it comes . . . What’ll happen will happen and invariably it happens alright . . . You’re going to get a bit of pain. You’re going to get one or two really, really bad experiences .. just say take it as it comes’* (Matthew).
*‘Acceptance is the big thing really . . . It just has to become part of your new routine, part of your life, until the day when it doesn’t have to be . . .* [be] *honest with yourself then it’s easier to deal with. Being in denial and trying to think that you don’t need it .., in fact makes it worse because being in a bad mood or a low mood it just makes it harder’* (Molly).

### Category 3: The necessity of cannulation for haemodialysis forces coping

Cannulation has to be completed to keep patients alive: ‘*The be-all and end-all is that I have to have dialysis so by hook or by crook .. I’ve got to get the needling done*’ (Michael). This meant: ‘*it can’t be escaped . . . it’s just something that you got to have, so. . . inevitable isn’t it so . . . it’s the needle isn’t it, you can’t escape it, it’s there*’ (James).

The necessity of cannulation motivated participants to get through the procedure (See Table 2 for additional quotes), although this was not always positive: ‘*You have to force yourself three times a week to go to somewhere where you know you’re going to get hurt, it’s essentially like a form of torture*’ (Clare).

Some participants experience haemodialysis and cannulation for many years. This made cannulation harder to cope with some reflecting that it will always be part of their life (see Table 2 for additional quotes). This category is further explained in three sub-categories: stoicism, acceptance and being able to contribute to cannulation.

Stoicism helps participants get through cannulation: ‘*I was accommodating the distress as one would accommodate pain . . . well it depended on one’s stoicism*’ (George).

They often use euphemisms to describe this (e.g. ‘*Grin and bear it*’ (Penny, Jessica, Rose, Nicholas), ‘*Grit your teeth*’ (Michael, Rose, James, Charlotte, Jessica)) as they ‘*just try and get myself through it*’ (James). They describe having to ‘*stand the pain*’ (Adam, George) or ‘*try and ignore it*’ (Adrian), as they have to ‘*learn to try and be like a grownup with needles*’ (Deborah). When using stoicism to cope, they need time before the cannulation ‘*to build myself up for it before I get there . . . not as much physically but mentally I would say*’ (James). A few participants suggest getting through cannulation makes them feel brave or strong: ‘*Err . . . just facing one of my worst fears . . . it makes me feel safe. That means I’m alive, you know . . . I’m going through it and I feel brave that I can do it*’ (Emma).

Participants describe accepting cannulation into their everyday life, where it becomes: ‘*part and parcel of me life now so yeah, I just take it as it comes*’ (Adam). The necessity of cannulation drives this acceptance: ‘*You have to accept it* [cannulation], *don’t you, because it’s part of the process, you can’t exactly miss them out . . . accepting really is the word*’ (Sarah).

However, although participants accept unpleasant cannulation, cannulation does not become better (see Table 2 for additional quotes).Being able to be active and contribute to the cannulation process also helps them cope and feel empowered: ‘*It made me feel like I was helping somebody and you feel like you’re speeding up the process . . . rather than just sitting there doing nothing and then saying ‘please help me’, that’s no good, that’s not the way that I do things, so yeah*’ (Simon).

Participants recognise as they are cannulated by healthcare professionals: ‘*there’s no way you can control your needling*’ (Matthew); thus, this was about feeling: ‘*part of the process, that it’s something you’re doing together*’ (Sarah). However, a few describe how they feel pressurised to contribute more than they were willing to: ‘*I’d definitely say I felt pressure . . . I’ve expressed that I don’t want to do it and they should respect that*’ (Molly).

### One final word from participants

Although cannulation is unpleasant, overwhelmingly participants clarify that they do not blame cannulators, recognising cannulation is difficult for cannulators too: ‘*It’s not always easy. You’ve got to .. you’ve got to look at it from their side. They can hit the vein, but they go through it go to the side of it or, you know . . . to be honest, I’d hate to be on the other side and try and needle some of those*’ (Daniel). They express felt gratitude towards the cannulators for being able to provide their haemodialysis and cannulation: ‘*Thank you, because I think they do a lovely job. I can’t do my own and I’m always extremely grateful that they’ve done it and managed to get it in . . . I think a thank you is what I’d like to say more than anything*’ (Penny).

## Discussion

Analysis of the semi-structured interviews highlighted that coping with cannulation is dynamic, where provision or absence of specific components facilitate or hinder coping. Three categories describe the components that facilitate coping with cannulation, which for the purposes of this discussion will be discussed in turn.

The first category, ‘Trying to make cannulation more comfortable’ highlights the importance of avoiding and minimising the unpleasantness of cannulation. Individuals highlighted different strategies that helped them to achieve this (Supplemental Material 4), which they developed through trial and error, advice from individual nurses or local procedures. However, no strategy was perfect, working for some and not others. Thus, there was no universal strategy that made cannulation more comfortable for all. This highlights the importance of an individual approach to cannulation, where components that facilitate coping for that individual should be available and implemented. Many of these strategies also lack a solid evidence base, with further research needed, exploring the effectiveness of interventions and the best way to implement these strategies to optimise their benefit for individuals.

Cannulation needs individual assessment and care plans to optimise patients experiences and promote the use of strategies that make cannulation more comfortable, with initial cannulation training promoting this. This approach also links with the second category, where the individuality of the patient is highlighted. However, haemodialysis environments are not always conducive to individualised care, with studies often highlighting how patients feel they are on an ‘assembly line’ or ‘just a number’ (Allen et al., 2011; Mafara et al., 2016). Haemodialysis nursing teams must consider how they promote an individualised approach in a busy environment.

The second category emphasises the individuality and humanity of the patient, recognising how the relationships surrounding cannulation can affect this. Empathy was a key part of maintaining patients’ individuality and humanity. This was about being understood by others, which had the benefit of not feeling alone through the cannulation. Empathy is different from sympathy and compassion, where empathy is about feeling ‘with’ someone, not ‘for’ them, with an affective dimension, ‘sharing another’s mental state’, a cognitive dimension, understanding what another is feeling, and a motivational dimension, where there is motivation to help another (Read, 2019). Trust was also critical to the relationship between the cannulator and the patient, with participants describing significant situations where trust had been lost. Allen et al. (2011) found that adversarial relationships developed in haemodialysis settings between healthcare professionals and patients, which reflected a lack of trust. Adversarial relationships can be avoided by using empathy to build relationships and promote trust (Allen et al., 2011). Cannulators need to understand what patients need to support them through cannulation, by understanding empathy and how to build trusting not adversarial relationships. Currently, training for cannulation often focusses on technical aspects to ensure a safe needle insertion. Training needs to start to incorporate understanding of patients’ experiences, facilitating empathy and creating understanding of how to build trust, which are key tenets of person-centred care.

The third and final category identifies how ‘The necessity of cannulation for haemodialysis forces coping’. In this category, the context of haemodialysis is important. This context acts as a motivator for coping, albeit forced due to its life-sustaining nature, activating stoicism to facilitate coping. Stoicism is defined as ‘silent endurance’ in response to distress or pain, including an ‘indifference to pleasure or pain’ with a ‘decreased willingness to complain’ (Moore et al., 2012). In healthcare, stoicism can be considered positive, creating resilience and preservation of self in difficult circumstances, but it can also be negative, denying the individual’s suffering (Moore et al., 2012; Pathak et al., 2017). A consequence of promoting stoicism is that interventions that may improve noxious symptoms are ignored and not utilised (Moore et al., 2012; Pathak et al., 2017). This was evident in earlier categories where interventions to make cannulation more comfortable were not implemented consistently, with participants using stoicism when they had nothing else to help them cope. There needs to be consideration as to the tools patients and nurses have available to them to support the patient through cannulation without relying on stoicism.

Acceptance is one tool to facilitate constructive coping, which is *‘the active embracing of subjective experience, particularly distressing experiences. The idea is not merely to grudgingly tolerate negative experiences but to embrace them fully and without defence*’ (Herbert and Brandsma, 2015: p.64). In opposition to stoicism, acceptance involves reaching a status quo with a new life situation often in response to difficult experiences or circumstances (Herbert and Brandsma, 2015). Therapies that promote acceptance, including cognitive behavioural therapies, acceptance and commitment therapies, behavioural activation and mindfulness (Herbert and Brandsma, 2015), could be utilised to promote acceptance of cannulation, with consideration whether cannulators could be equipped to support patients through cannulation with these approaches.

Recommendations and guidelines on cannulation for haemodialysis are sparse, with most focussing on vascular access (Aitken et al., 2025; Lok et al., 2020; Schmidl et al., 2018). There is one set of national cannulation recommendations (Fielding et al., 2018) that identify consistencies in cannulation practice through consensus, but also highlight a lack of evidence for cannulation practice. Understanding patients’ experiences of cannulation for haemodialysis is one of these key gaps. This study highlights the distress caused by cannulation for haemodialysis with potential solutions to minimise this distress, which are relevant to future guidelines and recommendations. Our study highlights the unique context of cannulation for haemodialysis, being a repetitive and frequent procedure for a long period of time, linked to a life-sustaining treatment. However, cannulation is a noxious procedure in other non-haemodialysis contexts (Welyczko, 2020), with our findings worthy of consideration.

### Implications

Cannulation for haemodialysis is an unpleasant procedure that patients endure regularly for long periods of time. Cannulators and educators, future guidelines and research need to focus on improving how patients experience this procedure. The results of this study indicate how this can be achieved, using various components to reduce and counteract the unpleasantness. Various strategies may improve individual experiences. Individualised assessment and care planning can facilitate their consistent application. Haemodialysis cultures and training need to consider how to make space for this individualise approach in a busy environment. Empathy and trust are key to preserving patients’ humanity and empathy during cannulation and optimising their experience. Cannulators need to consider how they facilitate and promote this throughout cannulation. Although stoicism is one component that facilitates coping with cannulation, cannulators and educators, future guidelines and research need to consider how to minimise the use of stoicism and promote acceptance, improving patients’ experiences of this procedure.

### Strengths and limitations

The use of CCA, intensive interviewing and theoretical sampling facilitated depth to our findings, enabling response to new insights and creating theoretical sensitivity, where the developing analysis was explored during interviews. The diversity in the sample was a strength, promoted through purposive and theoretical sampling, and recruiting participants whose first language was not English. The contribution of patient representatives throughout the study increased the authenticity of findings and assisted reflexivity.

This study was conducted across two renal centres, aiming to explore a breadth of cannulation experience in different centres. However, findings may not reflect experiences in all renal centres, particularly those with differing demographics and proportion of AV access. This study was conducted during the COVID-19 global pandemic. This may have influenced findings. The use of remote interviews changed the influence of the researcher on the interview, with a loss of non-verbal cues and increased use of verbal prompts, on occasions interrupting the flow of the interview.

## Conclusion

Coping with cannulation for haemodialysis was facilitated by various components of the cannulation. Patients attempted to make cannulation more comfortable through familiarity, minimising pain and unpredictability and reducing anxiety. Various strategies facilitated individuals to achieve this, but there was no universal strategy what worked for all. Cannulation requires individual assessment and care plans, with haemodialysis nursing cultures and training of cannulators promoting an individualised approach to cannulation. The strategies need further research to evaluate and explore their effectiveness. Recognising the patient’s humanity and individuality through empathy creates trust in the cannulator, improving patients’ experiences of cannulation. The context of haemodialysis made patients’ experiences of this type of cannulation unique and forced coping. Coping was facilitated by stoicism, acceptance and being able to contribute to cannulation. However, stoicism appeared to create a barrier to improving patients experiences of cannulation for haemodialysis. Therefore, focus needs to be on promoting acceptance, with training and research to develop interventions to facilitate, and equip cannulators to support this. These findings can be used to guide future guidelines and recommendations, both for haemodialysis and other cannulation procedures.

**Key points for policy, practice and/or research**

• Cannulation for haemodialysis is a distressing procedure for patients, associated with noxious symptoms.
• Patients use various strategies to try to make cannulation more comfortable by familiarity, minimising pain, increasing predictability and reducing anxiety.
• Preserving patients’ humanity and treating patients as an individual which is promoted by empathy and trust, makes cannulation easier to cope with.
• The necessity of cannulation for haemodialysis forces patients to cope with cannulation for haemodialysis, using stoicism, acceptance and contribution to the cannulation procedure.

## Supplemental Material

sj-docx-1-jrn-10.1177_17449871261466773 – Supplemental material for What influences patients’ experiences of cannulation for haemodialysis performed by nursing staff? A qualitative interview study Supplemental material, sj-docx-1-jrn-10.1177_17449871261466773 for What influences patients’ experiences of cannulation for haemodialysis performed by nursing staff? A qualitative interview study by Catherine Fielding, Sarah Brand, Louise Bramley, Maarten Taal, Caitlin Sorrell, Nicholas M Selby and Heather Buchanan in Journal of Research in Nursing

sj-docx-2-jrn-10.1177_17449871261466773 – Supplemental material for What influences patients’ experiences of cannulation for haemodialysis performed by nursing staff? A qualitative interview study Supplemental material, sj-docx-2-jrn-10.1177_17449871261466773 for What influences patients’ experiences of cannulation for haemodialysis performed by nursing staff? A qualitative interview study by Catherine Fielding, Sarah Brand, Louise Bramley, Maarten Taal, Caitlin Sorrell, Nicholas M Selby and Heather Buchanan in Journal of Research in Nursing

sj-docx-3-jrn-10.1177_17449871261466773 – Supplemental material for What influences patients’ experiences of cannulation for haemodialysis performed by nursing staff? A qualitative interview study Supplemental material, sj-docx-3-jrn-10.1177_17449871261466773 for What influences patients’ experiences of cannulation for haemodialysis performed by nursing staff? A qualitative interview study by Catherine Fielding, Sarah Brand, Louise Bramley, Maarten Taal, Caitlin Sorrell, Nicholas M Selby and Heather Buchanan in Journal of Research in Nursing

sj-docx-4-jrn-10.1177_17449871261466773 – Supplemental material for What influences patients’ experiences of cannulation for haemodialysis performed by nursing staff? A qualitative interview study Supplemental material, sj-docx-4-jrn-10.1177_17449871261466773 for What influences patients’ experiences of cannulation for haemodialysis performed by nursing staff? A qualitative interview study by Catherine Fielding, Sarah Brand, Louise Bramley, Maarten Taal, Caitlin Sorrell, Nicholas M Selby and Heather Buchanan in Journal of Research in Nursing
