# Supplementary material for: What influences patients’ experiences of cannulation for haemodialysis performed by nursing staff? A qualitative interview study
Source: J Res Nurs. 2026 Sep 18:17449871261466773. Online ahead of print. doi: 10.1177/17449871261466773 (PMC13588961; doi:10.1177/17449871261466773)
Supplement: Supplementary file 1 [file sj-docx-1-jrn-10.1177_17449871261466773.docx]

Concepts that Guided Theoretical Sampling

During the purposive sampling phase, the following concepts were identified as requiring further exploration:

- Experiences of patients who did not experience cannulation difficulty
- The effect of social isolation or social interaction during haemodialysis affected experiences of cannulation
- The effect of starting haemodialysis with a central venous catheter on patients’ experiences of cannulation of arteriovenous access
- The effect of limited communication and sensory deficits on patients’ experiences of cannulation for haemodialysis

Therefore, we recruited participants in theoretical sampling phase with the following characteristics:

- Patients who had no record of experiencing cannulation difficulties
  - Patients who had cannulation difficulties were interviewed during the purposive sampling phase
- Patients who dialysed in single rooms and small satellite units
  - Those dialysing in large haemodialysis units were interviewed during the purposive sampling phase
- Patients who started haemodialysis with a central venous catheter and then moved to arteriovenous access
  - Patients starting haemodialysis with an arteriovenous access were interviewed during the purposive sampling phase
- A patient who did not speak English fluently
- A patient who was blind
