# Supplementary material for: What influences patients’ experiences of cannulation for haemodialysis performed by nursing staff? A qualitative interview study
Source: J Res Nurs. 2026 Sep 18:17449871261466773. Online ahead of print. doi: 10.1177/17449871261466773 (PMC13588961; doi:10.1177/17449871261466773)
Supplement: Supplementary file 2 [file sj-docx-2-jrn-10.1177_17449871261466773.docx]

Supplementary Material 1: Initial Interview Questions

**Section 1: Your experience of cannulation**

1) *We are going to start the interview by talking about the needle insertion at the start of your haemodialysis treatments. Do you understand what I mean by needle insertion?* (Find out what terms works for them and use that throughout the interview – needling has been used consistently throughout but can be replaced by whatever term the patient uses).
2) *I wanted to hear about your needling at start of haemodialysis and what it is like for you. Can you tell me a bit about this?*

If the interviewee responds with mono-syllabic answers / struggles with this, probing questions can be used to draw this out, if needed:

- In your opinion, what is good or bad about your needling?
- In your opinion (do not repeat if already stated), what do you like / not like about your needling?
- How do you feel about your needling? How does it make you feel?
- Some people find it easiest to start at the beginning and talk it thought to the end, so I wondered if you could describe your needling starting from the beginning (questions can then be asked about how they felt at key point)?

The interviewee will also be asked to expand on points raised in response to these questions.

3) *Some people talk about having problems with their needling. I wondered if you’ve had any problems with your needling recently?*

If the interviewee responds with mono-syllabic answers / struggles with this, examples of problems getting the needles in at the start of treatment , like some people experiencing bruising or pain from the needles, can be used.

*How about in the past / in the beginning / at the start of being cannulated – did you have any problems then? Have you ever had any problems with your needling?*

If you have had problems:

- Could you describe these to me? / Can you explain what happens?
- Did the problems happen recently or a long time ago?
- Did the problems only happen once or do they happen regularly? Does it come and go? Is it common? Are you still getting problems now?
- How did they make you feel?
- Was there any reason why you thought you go these problems? Did anything change to make this better?
- Does anything make this better / prevent this happening again?

If you’ve not had problems, is there any reason why you think you’ve not had problems?

- Is there something about your access that means you don’t get problems?
- Do things always go smoothly or do you get hiccups but don’t see it as problems / cope with it / manage it?
- Is there anything else that you think helps prevent problems with your needling?

4) *Some people have told us that needling varies from session to session and over longer periods of time. From your perspective is the needling always the same or does it change?*

If it changes:

- How does it change?
- Does it change over short periods or longer periods?
  - E.g. day-to-day, week-to-week, month-to-month or year-to year?
- What changes about the needling? What is different about it? Does it get better or worse?
- Is it now better or worse than it was in the past?
- Is there any reason why you think it changes?
- How does it make you feel to have it vary / change?

If it does not change, is there anything that keeps it consistent / the same?

- Is what makes it consistent, good or bad, in your opinion (i.e. is it consistently good or consistently bad)?
- Do you think there is any reason why it is consistent with you?
- How do you feel about it being consistent?

**Section 2: What influences your experience of cannulation**

5) *Is there anything you can think of that makes a difference about how you feel about your needling?*

If so:

- What makes it feel good or bad?
- What makes it different?

The interviewer can explore concepts that have previously been mentioned by the interviewee. For example, if the interviewee has raised pain previously, the interviewer may ask what makes it more or less painful, here or earlier in the interview. This may also lead to the specific questions in the remainder of this section.

6) *Some people say they worry about having their needles put in for haemodialysis. However, we are not sure if the applies to everyone. Do you feel relaxed or worried when the needles are put in?*

Probing questions can be used to draw this out, if needed:

- Do you ever worry about whether the needles will go in OK or do you always feel relaxed about this?
- Is there anything that makes you worry about your needling?
- Is there anything that makes you feel relaxed about your needling?
- Does how much you worry or feel relaxed vary between different days / sessions / needling(s)?
  - What then makes you feel more or less relaxed on different days?

If you feel relaxed:

- What (helps) makes you feel relaxed?
- Do you always feel relaxed or does it vary?
- Is there anything that makes you feel more or less relaxed?
- Is there anything that you feel helps you to stay relaxed?
- Do you think being relaxed makes a difference to your needling?

If you feel worried:

- What are you worried about?
- Are you always worried about your needling or does it vary?
- Is there anything that makes you feel more or less worried?
- Do you think worrying makes a difference to your needling?

7) *I would like to ask about how safe you feel during the needle insertion. What sort of things make you feel safe or unsafe during the needle insertion procedure?*

- Is there anything that makes you feel like everything will be OK?
- Is there anything that makes you feel scared or fearful?

Probing questions can be used to draw this out, if needed:

- What might make you feel more or less safe?
  - Does it vary day to day, with how you feel?
- Is there anything that makes you feel more or less safe?
- Do you always feel safe / unsafe or does it vary?
- Is there anything that makes feeling safe / unsafe vary?
  - Is there anything that makes it better or worse?
- Does feeling safe / unsafe make a difference to your needling?

8) *I now would like to hear about whether you feel protected / looked after or vulnerable during the needling and what helps you feel this way.* (‘Looked after’ can be used as an alternative to protected if the interviewee feels more comfortable with this term. If the interviewee struggles with the term vulnerable, the interviewer can explain it is about feeling ‘exposed’ or ‘under threat’ and that this is often about whether they feel ‘at risk’.)

*What makes you feel protected or vulnerable during the needle insertion procedure?*

- Is there anything that makes you feel protected / looked after / cared for during the needle insertion?
- Is there anything that makes you feel vulnerable / exposed / at risk of harm?
- Do you always feel protected or vulnerable or does it vary?
- Is there anything that makes feeling vulnerable / protected vary?
  - Is there anything that makes it better or worse?
- Does feeling protected or vulnerable make a difference to your needling?

9) *Some people have stated it is important for them to feel in control of their needling. (*If required examples can be given, like inserting their own needles, having information about their needling, knowing where they are going to put the needles or who puts the needles in).  *What does it mean to you to be in control of your needling?*

*Is it important for you to feel in control during the needling or do you like to release control of this to rely on someone else?*

Probing questions can be used to draw this out, if needed:

- Do you feel you are given the opportunity to be part of the needling process? How does this make you feel?
- Do you like to be involved in decisions about your needling or do you leave this to the person inserting the needles? How does this make you feel?
- Do you feel you get the information you need about your needling or do you feel like you need more information?
  - How about at the very beginning when you started needling?
- Are there any barriers to you having the control you want over your needling?
- Is there anything that helps you to be in control of your needling?
- Can you describe any times when you have felt in control / out of control of your needling? How did this make you feel?
- Does how much control you are given vary or is it consistent? If so, what causes this to vary?

10) *Some people have said that the person putting the needles in can make a difference to how they feel about needling. Does the person inserting the needles make a difference to how you feel about the needling?*

*Is it always the same staff members that out your needles in or do lots of different people do this?*

*Does this make a difference to how you feel about your needling?*

Probing questions can be used to draw this out, if needed:

- Does the person inserting the needles do anything to make you feel better / worse about your needling?
- Is there any person you like putting your needles in?
  - If so what do they do, say or how do they behave to make you feel like this?
  - Can you describe how they put your needles in?
- Is there any person you do not like putting your needles in?
  - If so, what do they do, say or how do they behave to make you feel like this?
  - Can you describe how they put your needles in?
- Do all the people who put you needles in treat you the same and do the needle insertion the same or does it vary?
  - How does this make you feel?

11) *Do you feel there is anything about you or your access that makes you feel the needling easier or harder?*

Probing questions can be used to draw this out, if needed:

- Do you feel your fistula / graft is good or has problems that make needling better or worse?
- Has your fistula or graft changed to make needling better or worse?
- Does how you feel emotionally before the needling make a difference to your needling? If so, does vary on different days / sessions / needling?

12) *I would now like to ask you about your needling technique. Do you know if you use buttonhole or rope ladder technique?* (If required, an explanation can be provided that talks about how buttonhole cannulation goes into the same site each time, whilst rope ladder goes into a different site each time. For the purposes of the interviews, area puncture is considered the same as rope ladder.)

*Have you experienced any other techniques?*

*Some people say it makes a difference to them which technique they use. How do you feel about this?*

- Do you feel like you get lots of problems due to your cannulation technique or is it relatively problem free?
  - If you do get problems, what are these problems and what is the impact on how you feel about your cannulation?
- Is there anything about your technique that you feel makes your needle insertion better? If so, what is this?

13) *How do you feel when changes are made to your needling?* (If required further examples of this can be given which can include changing the sites where the needles go, using a new access, having a different team of nurses look after you, changes to the needle size, changes to the blood flow rate or any other changes to the needling or how it is used for haemodialysis).

- Have you experienced any changes to your needling?
  - If so, what were these?
  - How did they make you feel?
- Are there any other changes that may make a difference to how you feel about your needling?

14) *Is there anything else that affects how you feel about your needling?*

**Section 3: What could improve your cannulation?**

These questions may occur earlier in the interview as concepts are uncovered. However, it is also important these questions are covered even if these concepts have emerged previously. These questions also bring the interview to a close and becoming more positive, focussing on what can make needling better rather than what the problems are. Therefore, the interviewer will ask these questions at the end, but can also summarise and signpost to earlier content in the interview and adapt to allow this.

15) *Is there anything (else) that you think could make needling better?*

Probing questions could include, if required:

- Is there anything that would make you worry less?
- Is there anything that would make you feel safer?
- Is there anything that would make you feel less vulnerable?
- Is there anything that would make you feel more in control?

This question could link to issues they have raised earlier, asking the interviewee what might improve issues raised.

16) *Is there anything (else) you would like to change about your needling or anything good practice you’d like to highlight is helpful to you when going through needling?* This can be realistic or unrealistic
17) *The interview is now coming to a close. Is there anything else you would like to say about your cannulation / needle insertion?*
18) *Do you have any further questions or comments before we finish the interview?*
