# Supplementary material for: What influences patients’ experiences of cannulation for haemodialysis performed by nursing staff? A qualitative interview study
Source: J Res Nurs. 2026 Sep 18:17449871261466773. Online ahead of print. doi: 10.1177/17449871261466773 (PMC13588961; doi:10.1177/17449871261466773)
Supplement: Supplementary file 3 [file sj-docx-3-jrn-10.1177_17449871261466773.docx]

Supplementary Material 2: Changes made to Interview Questions

Changes made after 16 interviews

Following the first 16 interviews, questions around safety, vulnerability and the AV fistula / graft (Qu 7, 8 and 11 in the initial interview guide) were removed as participants did not understand or relate to these questions. Question 13 in the initial interview guide, asking about changes made to their needling, was merged with Question 4 (in both interview guides) as participants had found the two separate questions repetitive. Minor edits were made to existing questions to reflect what worked and did not work in interviews (these changes have not been recorded). The order of questions was also altered to reflect the expected flow of the interview. The altered and added questions are shown below:

**Question 5 (new question)**

*Do you feel the needling affects your haemodialysis at all or does it make no difference to it?*

If it does make a difference:

- How does it affect your haemodialysis? What difference does it make?
- Does it always make a difference or does this vary?
- Has this changed over time?
- How does this make you feel:
  a. About your haemodialysis?
  b. About your needling?

If does not make a difference:

- Does the needling make any difference to how you feel about your haemodialysis or does it make no difference what so ever?
- How do you feel the needling is separate / distinct / independent to the haemodialysis?

**Question 6 (new question)**

*I was wondering if you can remember how prepared you felt for needling when you first had it. Did this make a difference to how you felt about your needling at the time?*

*Does this make a difference to how you feel about your needling now?*

Probing questions to draw this out could include:

- Can you remember how your very first needle insertion felt?
- Do you think you were ready for needling when it first happened? How did this make you feel?
- Did you know what to expect for your first needling? How did this make you feel?
- Did you receive any information about your needling before it happened? How did this make you feel?
- Is there anything that could have made you feel better prepared for needling or do you think you had everything you needed / that could be done?
- Did you first needle insertion coincide with your first haemodialysis session? Do you feel this made a difference to how you felt?

**Question 8 (new question)**

*Some people say that they find the needling painful. I wondered whether you find the needling painful or not?*

If it is painful:

- Could you describe to me what the pain is like?
- Has it always been like this or has this changed over time?
- How does the pain make you feel?
- Have you noticed whether anything makes it more or less painful?
- How does that make you feel about your needling?

If it is not painful:

- Is there anything that you think makes it not painful?
- Has it always been like this or has it changed over time?
- Is always not painful or can it be painful sometimes?
- How does that make you feel about your needling?

**Question 10 (new question)**

*Some people say that the people around them can make a difference to how they feel about their needling. Do / Would other people being around you during the needling make a difference to your needling, or do they make no difference?*

Probing questions to draw this out can include:

- Do you have other patients around you / in the same area as you during the needling? How does this make you feel?
- Does anyone sit with you during the needling? How does that make you feel?
- Do other people make a difference to how you feel about your needling?
- Do you feel other people make needling easier or harder or make no difference? What is it that other people do to make it better / worse / different?

**Question 11 (new question)**

*Some people have said that the environment can make a difference to how they feel about their needling. Does the environment or the atmosphere around you during your needling make a difference to you or not?*

Probing questions can be used to draw this out, if needed:

- Does where the needling happens make a difference to you?
- Does the atmosphere on the unit affect your needling at all? If so, how does it make a difference?
- Do you dialyse on a large or small unit? Does this make a difference to how you feel about your needling?
- Is there anything you like or don’t like on the unit that makes a difference to your needling?

**Question 14 (was Question 12) – extra text and questions added**

‘*I would now like to ask you about buttonhole and rope ladder needling. Do you know which technique you have?*

At this point the interviewer will check understanding with the interviewee about what buttonhole and rope ladder means to them and provide an explanation where needed.

- Buttonhole goes into the same site each time. They normally remove the scab from the previous needling before inserting the needle. They develop a track using sharp needles and then move you over onto blunt or dull needles.
- Rope ladder goes into a different site each time and the needle sites move up (and sometimes down) your arm. Needles sites are meant to cover as much of fistula / graft as possible. (For the purposes of the interviews, area puncture is considered the same as rope ladder.)

*Some people say it makes a difference to them which technique they use. Does this make a difference to you?*

Probing questions can be used to find out about their opinions on their technique and the one they do not use (*= alter question according to which technique they use):

- How does it feel to be needled in the same place / different place* each time?
  - How would you feel if you were needled in the same place / different place* each time?
- Does using dull / sharp needles* make any difference to how you feel about your needling?
  - How would you feel if they started using dull / sharp needles* on your fistula?
- Review explanation of their technique*
  - Is there anything you like / dislike about your needling technique?
- Review explanation of other technique*
  - Is there anything you like or do not like about the idea of the other technique?
- How do you feel about the appearance of your fistula?

**Question 17 and 18 (was Question 16, now split into two questions)**

17) *Is there anything (else) that you think could make needling better for you?*

This question could link to issues they have raised earlier, asking the interviewee what might improve issues raised.

18) *Is there anything (else) you would like to change about your needling?* *This can be realistic or unrealistic.*

There were also emerging concepts that were difficult to ask participants about in direct questions, but through theoretical sensitivity detected the appearance of these concepts and then explored these further. These concepts were:

- Empathy from the person inserting the needle
- Being treated with humanity and as an individual
- The unpredictability of needling
- Acceptance of the procedure

Changes made after 21 interviews

This was a minor change to the interviewing guide to reflect one further emerging concepts. This added one further question, which became question 6:

*‘Some people have mentioned that the frequency of needling for haemodialysis alters how they feel about their needling. I wondered how you feel about having the needling X (insert how often they dialyse) times a week?*

Probing questions to draw this out can include:

- What difference do you think the frequency of your needling makes or does it make no difference at all?
- Do you think having the needling this frequently (x times a week) makes a difference to your needling or how you feel about it?
- If it was only a one-off procedure or less frequent, would you feel differently about your needling?
- If you’re needling was more frequent (e.g. every day), would this change how you felt about it?
- Does anything make the frequency of needling easier or harder to manage?’

One further concept was added to explore further without a direct extra question, but to use theoretical sensitivity to identify when it appeared and then probe deeper into its meaning. This was stoicism.
