# Supplementary material for: What influences patients’ experiences of cannulation for haemodialysis performed by nursing staff? A qualitative interview study
Source: J Res Nurs. 2026 Sep 18:17449871261466773. Online ahead of print. doi: 10.1177/17449871261466773 (PMC13588961; doi:10.1177/17449871261466773)
Supplement: Supplementary file 4 [file sj-docx-4-jrn-10.1177_17449871261466773.docx]

Strategies that Made Cannulation more Comfortable for Individuals

Throughout interviews, participants shared strategies that made cannulation more comfortable for them. This supplementary material shares the detail of these strategies, which may be useful for cannulators to use with individual patients, researchers to investigate their effectiveness in future studies and clinical recommendations to consider. As mentioned in the main article, none of these strategies worked for all participants and none have been fully evaluated in research.

The table below describes these strategies, providing a description of each strategy and quotes form participants to evidence their perspective on the strategy. Participants often felt these strategies were implemented inconsistently, which is highlighted in descriptions and quotes. Each strategy related to a sub-category within category 1, ‘Trying to Make cannulation more Comfortable’.

| Strategy | Description of Strategy | Sub-Category | Participant Quotes from Interviews Describing the Strategy |
| --- | --- | --- | --- |
| Being provided with information on cannulation | Participants described how having more information on their cannulation would help them develop familiarity and make cannulation easier. Before they started cannulation they felt being provided with written and verbal information would have helped them prepare for this. Once they were on haemodialysis, they felt changes and problems were not explained to them and they often became frustrated with this. Some participants indicated that they initially refused information, but then later regretted this as cannulation was inevitable. These descriptions indicated information needed to be given in a sensitive manner. | Familiarity makes cannulation less alien | *‘At first I was very, very nervous … it’s not knowing … it’s the unknowing … so a lot of information would have been quite acceptable, better. I think it would have eased the apprehension.’* (David) |
|  |  |  | *‘I don't really feel I'm give information, unless I force it. I sometimes feel like the opinion is I can't cope with that information or it’s not useful to give me any information. Makes me a bit frustrated, to be fair. Because I’d like to think I was…hmm…interested enough in my – what’s going on with me - and intelligent enough to take information on-board. But I don't think that’s always taken into account.’* (Sarah) |
|  |  |  | *‘When I got letters to go to other workshops and things, I didn’t go. So when other people went and had a look around the dialysis unit, I missed out because I was too frightened … If I could have sat down and spoken to someone at that point and they could have explained to me in not frightening terms, that would have been so helpful.’* (Jessica) |

| Strategy | Description of Strategy | Sub-Category | Participant Quotes from Interviews Describing the Strategy |
| --- | --- | --- | --- |
| Injecting sub-dermal lignocaine prior to cannulation | Some participants found sub-dermal lignocaine injected prior to cannulation made the cannulation less painful. However, not all found the lignocaine beneficial because it didn’t always work, it involved another injection / needle or it was painful to have the lignocaine. | Avoiding or Minimising Pain from Cannulation | *‘Lignocaine was a godsend! … At the very beginning, I was wanting Lignocaine all the time, because I knew it numbed some of that pain.’* (Deborah) |
|  |  |  | *‘If it doesn’t settle in straightaway I can still feel it, so it obviously defeats the object.’* (James) |
|  |  |  | *‘You're still needled for the Lignocaine … and my preference is not to have needles full stop’* (Simon) |
|  |  |  | *‘When they put that first in that feels the same like burning. Yeah, like a nettle sting sort of.’* (Adrian) |
| Applying topical anaesthetic cream prior to cannulation | Some participants found topical anaesthetic cream applied 30-60 minutes before cannulation reduced the pain from cannulation. However, again this did not work for all, with some finding it ineffective and some developing allergies to the cream. | Avoiding or Minimising Pain from Cannulation | *‘And the .. erm .. I’ve got this cream now what you put on and it sort of numbs it … And that’s made it a lot easier.’* (Michael) |
|  |  |  | *‘The first couple of times I had the numbing cream and I can't really say that did anything.’* (Molly) |
|  |  |  | *‘I’m allergic to the cream. They had to stop using it’* (Jessica). |
| Using documentation to communicate how to cannulate individual AV access | Participants discussed how nursing teams used documentation to communicate about how to cannulate them. They found this reassuring, but often felt frustrated as cannulators did not always look at this documentation prior to cannulating them, which led to problems during the cannulation. | Making Cannulation more Predictable: Getting the Needles in easily | *‘Whoever’s doing it, it’s written in the notes that it’s either shallow or deep or whatever’* (Charlotte). |
|  |  |  | *‘It’s supposed to be written down in front of them. All they have to do is read it, you know, it’s the instruction, they have to read the instructions and do it … I have to make sure, I tell them to write things down on my prescription … they don’t!’* (Ben) |
| Participants learning the best way to needle their fistula | Some participants learnt how they were cannulated successfully, so they could then communicate this to other cannulators prior to cannulation. However, they often became frustrated if the cannulator did not listen to them. |  | *My graft is very shallow and sometimes when I say to a nurse who hasn’t done it before .. I’ll say to them look it’s very shallow and it jumps … I always explain that so that they understand … [audible sigh] I mean it’s inevitable, it’s human nature but if it’s the first time, they’ve gone ‘oh oh it is shallow isn’t it?’ I think yeah, I told you that.’* (Avril). |
| Applying a tourniquet immediately prior to and during cannulation | Some participants found applying a tourniquet prior to the cannulation made the cannulation more successful and easier. However, this did not work for everyone and not all cannulators were confident using a tourniquet. | Making Cannulation more Predictable: Getting the Needles in easily | *What is it – tourniquet? …* *‘it helps to us or the nurses to find the slot, yeah.’* (Graham) |
|  |  |  | *‘I understand why some people don't, because it’s I guess the way they were taught is just different … I wouldn't want someone to use a tourniquet and not know how to use it, because you can make things a lot worse if you don't do it properly, you know.’* (Clare) |

| Strategy | Description of Strategy | Sub-Category | Participant Quotes from Interviews Describing the Strategy |
| --- | --- | --- | --- |
| Use of point of care ultrasound to assist with cannulation | Some participants described their experiences of the cannulator using a bedside ultrasound scanner to help them find the vein and insert the needle. They felt this led to a more successful cannulation. The ultrasound scanner was generally only needed when the AV access was new, or. Once the cannulation was established, the ultrasound scanner was not required unless they experienced further problems during cannulation. However, not all cannulators could use the ultrasound scanner which either led to problems during the cannulation if it was not used or delays to the cannulation whilst they waited for a cannulator who could use this. | Making Cannulation more Predictable: Getting the Needles in easily | *‘And so they got that ultrasound thing out and they scanned my arm, found the vein and then went in like that and it worked.’* (Adrian). |
|  |  |  | *‘I had to have it scanned … for them to find it* [the vein] *again … then they found it and then they just did it from that.’* (Toby) |
|  |  |  | *‘But when I had that needler and that gave me the first blow, they didn’t have the scanner. So they didn’t learn how to initially do mine perfect first time because it didn’t have the scanner. Its just tings like that where maybe it would have bene prevented.’* (Nicholas) |
|  |  |  | *‘I needed the ultrasound machine to find my needle to put it in … and I had someone kind of get annoyed that I wanted them to use the machine saying they didn’t know how to use it and there was no-one there that knew how to use it, so I'm like ‘oh OK fine’.* (Clare) |
| Thinking differently about cannulation including trying to relax | Participants tried to actively change their mindset or the way they thought about canulation, which they felt helped reduce their anxiety about this. However, they did not find this easy to do and this took conscious effort. | Reducing Anxiety about Cannulation | *‘Be relaxed, because if not - it’s just a mind, just mind-set you know … If you think pain, pain, pain, then it’s pain .. if that I said that I’m happy I think that ‘Oh I’m happy’ .. If I’m sad and I’m thinking sadder things and if my life is upset as well’ (Tessa)* |
|  |  |  | *‘They usually say ‘relax your arm, relax your arm’ but when somebody’s putting a foreign body into your body, it’s .. erm . . not that easy is it?’ (Michael)* |
|  |  |  | *‘The objective is to trying* [sic] *not to tense up so that they can go through, but that’s also quite difficult with the fear of needles.’* (Michael) |
| Thinking about something else during cannulation | Participants tried to avoid thinking about cannulation whilst it was happening, using thoughts about something pleasant to achieve this. This was a distraction strategy that stopped them focussing on the unpleasantness of cannulation. However, this did not work for everyone. | Reducing Anxiety about Cannulation | *‘I just pretend I'm on an island with some sun somewhere and just look away … just pretend I'm somewhere else! [Laughs] Until it’s all over and then obviously you get the pain back and you're back in the hospital! [Laughs].’* (James) |
|  |  |  | *‘Think of something nice, as you don’t want to be here.’* (Rose) |
|  |  |  | *‘People kind of say ‘go to your happy place’ and its like I’m in my happy place while the needles go in and then it’s painful in my happy place! You know it’s not that easy.’* (Clare) |

| Strategy | Description of Strategy | Sub-Category | Participant Quotes from Interviews Describing the Strategy |
| --- | --- | --- | --- |
| Using breathing techniques during cannulation | Some participants described how breathing techniques helped manage their anxiety during cannulation. | Reducing Anxiety about Cannulation | *She told me to, she gave me some like breathing techniques to try, like deep breaths or slow, shallow breaths.’* (Steven) |
| Squeezing something during cannulation | One participant felt that squeezing something during cannulation helped reduce her anxiety. She often recommended this to other participants. | Reducing Anxiety about Cannulation | *‘I do have a little soft toy that my partner bought and I squeeze it when they're putting the needles in, so that kind of helps a bit … I've given it to some other people when they have been having troubles and they're kind of getting frustrated, I’ll offer my soft toy services to them to help kind of relieve some of that stress!’* (Clare) |
| Listening to music during cannulation | Some participants found listening to music helped distract them from the unpleasantness of cannulation. This was either music playing out loud in the clinical area or some listened via headphones. | Reducing Anxiety about Cannulation | *‘I will do my best to tune into the music and try and sing the music in my head. So the music is taking my mind off what they're doing, so I'm not 100 % concentrating on what they're doing, I'm trying to think of something else.’* (Penny) |
|  |  |  | *‘I don’t get anxious because I listen to my music.’* (Ben) |
| Chatting | The majority of participant found that chatting helped ease their anxiety and sometimes distracted them from cannulation. | Reducing Anxiety about Cannulation | *‘I think just general chit chat really … you start talking about something, you can have a bit of a laugh and a joke about it and you're easy before they put the needles in and then they put your needles in and it’s OK’*. (Bridget) |
|  |  |  | *‘I think the camaraderie, the way you approach people, the way if you’re not too clinical, if you approach us … somebody as a friend that makes a difference, if you start to make it clinical I think that’s when people start to get apprehensive.’* (Daniel) |
| Reading or watching something during cannulation | Either reading or watching something else helped distract participants from cannulation and ensured they did not focus on the unpleasantness of cannulation. | Reducing Anxiety about Cannulation | *‘It’s just a matter of just keeping your mind occupied. I just normally sit there with my phone and just go on the internet and just read my papers and that.’* (Martin) |
| Physical sensation elsewhere | One participant found using physical distraction helped distract from the unpleasantness of cannulation. | Reducing Anxiety about Cannulation | *‘I tend to pinch the inside of my thigh to confuse my nerves! … And it does sort of distract … it’s not concentrating on your arm and you're thinking about oh my leg – you sometimes forget that you're even being needled because you're thinking oh my leg hurts! It’s all very basic but it works for me.’* (Molly). |
